# Role of Metacognition Thinking and Psychological Traits in Breast Cancer Survivorship

**DOI:** 10.3390/bs10090135

**Published:** 2020-09-07

**Authors:** Jessica Ranieri, Federica Guerra, Dina Di Giacomo

**Affiliations:** Laboratory of Clinical Psychology and Psychoncology, Department of Life, Health and Environmental Sciences, University of L’Aquila, Via Spennati n.1, 67100 L’Aquila, Italy; jessica.ranieri@univaq.it (J.R.); federica.guerra@graduate.univaq.it (F.G.)

**Keywords:** breast cancer, metacognition, distress, fear of recurrence, cancer-specific psychological treatment

## Abstract

Longer survivorship is possible due to advances enabling early detection and treatment. However, cancer survivors are faced with prognostic uncertainty regarding survival, long-term symptoms, surveillance, and consequences of treatment. This study aimed at investigating emotional traits of women after breast cancer (BC) diagnosis from a three-year perspective of the disease. We intended to examine the emotional trend within longer survivorship after the primary treatment for BC. A sample of 72 women diagnosed with breast cancer (age range 30–55 years) was evaluated based on metacognition (Metacognitive Questionnaire—30 test), psychological distress (Psychological Distress Inventory test), anxiety, stress, and depression (Depression, Anxiety and Stress Scale—21 test). The data analysis applied was descriptive analysis, ANOVA, MANOVA, and ANCOVA comparing MCQ-30 variables and psychological traits (PDI, DASS-21). The results indicated positive recovery after primary care despite emotional fragility in survivorship owing to negative thoughts; correlations among metacognitive factors, anxiety, and distress not only confirmed the negative emotional pattern just after primary care, but also showed women regaining a positive emotional pattern in daily life. The survivors exhibited emotional fragility during certain specific points of time during the course of their survivorship. Based on our findings, the fear of recurrence and cancer-specific psychological treatment is a better framework to boost and improve clinical practice.

## 1. Introduction

Breast cancer (BC) diagnosis is a stressful event that negatively impacts the patient’s emotions and quality of life, triggering depression, anxiety, sleep disorders, and psychological and physical health-related problems [1,2,3,4,5]. Mental and physical problems, in addition to types of cancer treatment, such as surgery, radiotherapy, chemotherapy, and hormonotherapy, can affect the patient’s family life, work, social activities, and sexual functioning. However, recent studies highlighted that those diagnosed with BC at a younger age can exhibit emotionally resilient patterns and higher positive outcomes [6,7,8,9,10,11]. After active treatment (surgical and pharmacological intervention in primary care), young women tend to cope well with cancer-related stress and protect themselves against the negative effects of stress by lessening or absorbing the shock of the cancer diagnosis, the impact of aversive events, and of related life changes, thus improving mental health and treatment outcomes. The review of Seiler and Jenewein [11] suggested psychological resilience and post-traumatic stress growth as critical components of cancer-related care, highlighting their protective effect on survivorship. 

Advances that help early detection and treatment enable longer survivorship. However, cancer survivors face prognostic uncertainty about survival, long-term symptoms, surveillance, and consequences of treatment. Fear of cancer recurrence (FCR) is a significant distress-causing factor that affects a substantial number of patients with, and survivors of, cancer. Koch et al. [12] described FCR as being consistently associated with increased functionality impairment, psychological distress, stress response symptoms, and lower quality of life. Recent metacognition investigations seem to support this. Mutlu et al. [13] analysed metacognition in cancer patients in early and metastatic stages. Patients exhibit higher levels of negative metacognition in early stages and the lowest levels in the metastatic stage. The authors suggest the ambiguity of prognosis during the early stages to be a possible reason for this. Quattropani et al. [14] demonstrated the role of negative belief as a predictor of anxiety and psychological distress in patients undergoing chemotherapy. The authors showed the association between metacognition and emotional distress, verifying negative belief in particular as the predictor of negative anxiety, depression, and overall distress. Despite great interest in the psychological impact of diagnosis and treatment of BC, these findings are not exhaustive because most investigations were conducted while they were inpatients and not when they went back to daily life, to work, and to their own habits. Their social and affective community must be thoroughly inquired to boost their quality of life after the traumatic experience of cancer diagnosis and treatment. It could be interesting to investigate the relationship between personality traits and metacognition thinking over the time and how they change and influence wellness and quality of life of women winning life back. 

Our investigation aimed to evaluate the trend of emotional traits in screened women over a longer period, starting from the end of primary care. We intended to analyse the role of metacognitive thinking aspects in women after hospitalisation from an observational perspective, spanning over three years, involving different patients in four phases of their survivorship. Employing an observational study design, our goal was to analyse metacognition associated with depression, anxiety, stress, and signs of psychological distress among Italian BC women over a period of 36 months. We evaluated the psychological traits and metacognitive thinking about cancer-related experiences of women dealing with the related complex clinical path, even exploring the impact of aging on metacognitive thinking. 

## 2. Materials and Methods

### 2.1. Ethical Approval 

This study was approved by the Internal Review Board of the University of L’Aquila, Italy (Prot. N° 16,372/2019). 

### 2.2. Participants

Eligible participants included women diagnosed with BC, aged 38–55 years (mean age 48.3 years, sd ± 6.2), living in central and north Italy. Exclusion criteria included recurrent or metastatic cancer, psychiatric or neurological disturbances, and alcohol or substance abuse. Patients were approached for participating in the study at the Medical Oncology Division of S. Salvatore Hospital in L’Aquila (Italy). The study was focused on the female population in order to analyse homogeneous emotional influences and reactions. 

We contacted 82 eligible patients. Of these, 72 provided informed consent, and 10 did not agree to participate in the experimental protocol. Table 1 shows the participants’ demographic characteristics. 

Participants were eligible to enrol in the study if they were diagnosed with BC and followed primary care. Inclusion criteria included: (a) 35–55 years old (age), (b) female (gender), (c) no recurrence (second diagnosis), (d) diagnosis and primary treatment of breast cancer and, (e) I–III stage of cancer. The Tumor, Node and Mestastasis (TNM) classification of malignant tumours, a cancer staging system developed by the American Joint Committee on Cancer and the Union for International Cancer Control (UDIC), was used to classify the patients’ cancer stage by medical staff. 

### 2.3. Procedure 

Medical staff in the oncological division identified patients, who were then enrolled during subsequent follow-up appointments. Informed consent was obtained at the time of enrolment. Trained clinical psychologists, blind to the objectives of the study, conducted the psychological evaluations in a quiet, dedicated room. The duration of the evaluation was 40 min. Participants completed the measures during their scheduled follow-up appointments. Data were collected anonymously.

### 2.4. Measures 

#### 2.4.1. Sociodemographic and Clinical Variables 

Two types of patient information were collected. First, demographic data were provided via patient self-reports. We selected independent variables for inclusion in the analyses if they were characteristic of the age/life stage (e.g., having children, being employed, marital status) related to cancer.

Second, clinical data were obtained from the patients’ medical records regarding the breast cancer stage, treatment, and therapies.

#### 2.4.2. Psychological Tests

The experimental psychological battery was composed of self-reports evaluating emotional variables: anxiety, psychological distress, and depression, as well as metacognitive thinking. Participants took the tests after the clinical interview in individual sessions. 

The psychological tests used included the Psychological Distress Inventory (PDI) to assess distress, the Depression Anxiety Stress Scale (DASS-21) to detect the anxiety, stress, and depression dimensions, and the Metacognition Questionnaire (MCQ-30) to detect the beliefs about the patients’ own disease conditions. All tests was applied in the version adapted to the Italian population. 

*Psychological Distress Inventory* (PDI) [15]. This self-administered questionnaire measures the impact of psychological distress and related therapies. It is composed of 13 questions and responses are indicated on a Likert-type scale (five points). The standard score estimates the presence/absence of psychological distress to measure global distress. This test was administered only to the patient group. The inventory demonstrated good reliability (α = 0.86).

*Depression Anxiety Stress Scale* (DASS-21) [16]. The DASS is a clinical assessment that measures the three related states of depression, anxiety, and stress. It has 21 questions and takes about 3 min to complete. Each subscale measuring the emotional traits is composed of seven items. 

*Metacognition Questionnaire*-*30* (MCQ-30) [17]. The MCQ-30 is a brief multidimensional measure of metacognition and individual differences in a selection of metacognitive beliefs, judgments, and monitoring tendencies considered important in the metacognitive model of psychological disorders. Alpha reliabilities for the five subscales range from 0.72 to 0.89. The five subscales are: (1) positive beliefs about worry (POS), (2) negative beliefs about thoughts concerning uncontrollability and danger (NC), (3) cognitive confidence (assessing confidence in attention and memory, CC), (4) negative beliefs concerning the consequences of not controlling thoughts (NEG), and (5) cognitive self-consciousness (the tendency to focus attention on thought processes, CSC). In the present study, a validated Italian version of the MCQ-30 [18] was used to assess metacognitive beliefs. The results of the Italian MCQ-30, like the original version, indicated direct correlations between metacognitive factors (except for CSC) and state and trait anxiety, pathological worry, and obsessive–compulsive symptoms.

### 2.5. Study Design

The current study was observational and conducted to evaluate the prevalence of psychological traits within the BC population. 

Descriptive statistics for measures were calculated in order to analyse the psychological traits and metacognitive thinking of the target; ANOVA, MANOVA, and ANCOVA (followed by Tukey’s post hoc analyses) were conducted to detect the statistical significance of the overall differences across the psychological variables by stage of the disease and age. Then, Pearson *r* correlations were applied. All tests had matching psychological scores by demographic and clinical variables in order to verify their reciprocal influence. 

The data analysis was performed using SPSS, with a fixed α-value ≤ 0.05. 

## 3. Results 

Overall, 88% of eligible women completed the psychological evaluations, and 10 women declined to participate (no returned consent form). 

Table 2 shows the mean (and standard deviations) of the raw scores performed in the psychological tests.

Our statistical analyses were focused on the emotional experience of patients in the time period after the breast cancer diagnosis. MANOVA was conducted to compare their emotional status. Participants were divided into four subgroups according to the timeframe after diagnosis and primary medical treatments: T0 included patients in the time range of 6–11 months from diagnosis, T1 in the range of 12–18 months, T2 in the range of 19–24 months, T3 in the range of 25–36 months. The timeframes were representative of scheduled medical check-ups. MANOVA (5 × 4) was conducted comparing MCQ-30 variables, (3 × 4) on DASS-21, and then ANOVA one way on PDI with timeframes of evaluation. The statistical analysis showed a significant difference in negative beliefs between different timeframes (F(68, 3) = 7.0; η = 0.7; *p* > 0.01). Post hoc analysis (Tukey’s test) indicated lower scores at T0 (*p* > 0.01) and T3 (*p* > 0.01). 

No significant effects on PDI and DASS-21 were observed over time.

Subsequently, one-way ANOVA (5 × 2) was conducted comparing MCQ-30 variables (POS, NEG, CC, NC, CSC) and age groups (young and adult) (see Table 3). It showed a significant effect in the NEG index (negative beliefs concerning the consequences of not controlling thoughts) (F(68, 1) = 7.0; η = 0.7; *p* > 0.01). The younger group seemed more worried than the adults. There were no significant differences in the other MCQ-30 indexes. The findings indicate that adult patients are emotionally more controlled in the negative aspect, whereas the younger ones are frightened. 

One-way ANOVA (3 × 2) comparing DASS-21 indexes (anxiety, depression, and stress) by age groups revealed a significant difference in anxiety performance (F(68, 1) = 6.0; η = 0.6; *p* > 0.01): the younger group showed more anxiety than the adults.

Finally, statistical analyses were performed, classifying participants by the time from the initial diagnosis into four timeframes: T0 (6–11 months), T1 (12–18 months), T2 (19–24 months), T3 (25–31 months) according to the scheduled medical protocol follow-up. Table 3 shows the score data. ANCOVA (3 × 4 × 2) was conducted and comparing DASS-21 indexes, timing (T0, T1, T2, T3), and age groups revealed significant differences. 

**Figure 1:**
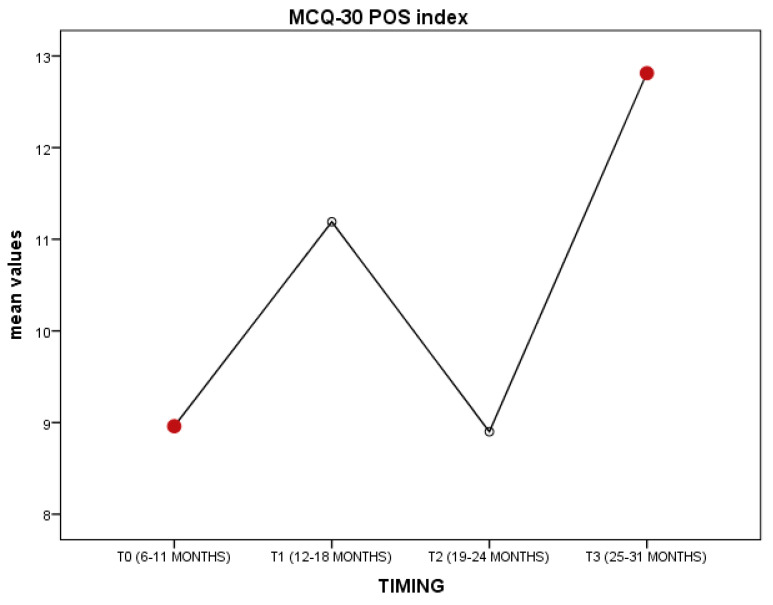
Representation of MCQ-30 POS index over time.

ANCOVA (4 × 4 × 2) conducted on MCQ-30 indexes, timing, and age was significant for POS variables (F(68, 3) = 3.5; η = 0.7; *p* > 0.02) covariated by timing; no significant covariance by age. Post hoc analysis (Tukey’s test) showed the POS variables to be lower in T0 (*p* = 0.01) than T3. However, although not significant, data showed the increasing trend of positive beliefs over time, statistically detectable in T3. The findings show that young patients were more worried right after the end of primary treatment and starting the post-treatment (even survivorship). However, though not significant statistically, as represented in Figure 1, T2 could be critical for patients; the POS variable tended to decrease and could be associated with fear of recurrence. 

This effect is interesting and reflects the fragility of women during survivorship: despite stress or depression, they tend to develop adaptive behaviour and live well, overcoming the BC diagnosis and treatment. However, it is harder to deal with everyday life in survivorship, overcoming the fear of recurrence. Table 4 shows Pearson correlation coefficients for the MCQ-30, DASS-21, and PDI.

The results presented in Table 3 show that most of the correlations between the MCQ-30 scores and the DASS-21 and PDI scores range from moderate to strong. 

In terms of potential relationships between negative metacognitive factors and emotions, the correlations between negative beliefs and anxiety, stress, and even distress scores were the highest; correlation with depression was moderate; even the beliefs about the need to control thoughts (NC) was moderately significant with anxiety; cognitive confidence (CC) showed a moderate correlation coefficient with the distress index.

## 4. Discussion and Conclusions 

This study investigated the relationship between the psychological traits and metacognitive thinking of women after BC diagnosis from a three-year perspective of the disease. Our purpose was to examine emotional and thinking changes in survivorship after the primary treatment for BC. 

Overall, our study revealed the positive adjustments patients are capable of making despite being diagnosed with this illness. Our participants brought to light the strong ability of women to be resilient by regulating their emotions and being flexible, dealing energetically with the different steps involved in clinical settings, surgery, and pharmacological treatments. Over the 3 years following diagnosis, the timeframes when they were most strongly impacted were around the 12th month after diagnosis and between 24 to 36 months post diagnosis. Several studies detected the negative psychological impact in older women expressed as depression, anxiety, and personality disorders [1,2]. Considering our previous findings [6,7,8], this study confirmed a positive emotional trend among young women, impacting their cancer-related experience over time. The women seemed to cope well and showed fragility during specific points of time of their survivorship, suggesting a targeted psychological treatment. Metacognitive processing of cancer-related experience shows how psychological coping works over the time. Mutlu et al. [13] highlighted higher negative thinking patterns regardless of the specific diagnosis just after the primary treatment and associated this with psychopathology, such as depression and anxiety. Our study confirmed the findings and the negative thoughts right after primary care, influencing psychological traits. However, our study also showed women going back to a positive thought pattern in their daily lives, improving psychological wellness. 

According to this study, two variables can be emotional prompts for being resilient: the early diagnosis and target lifetime. The early diagnosis of BC refers to applying, in medical protocols, more conservative and less invasive surgical and pharmacological intervention in primary care, preserving the patient’s health, and testifying for better prognosis. Diagnosis at an early age (<50) normally involves women being deeply involved in the family/work/affective perspectives, and they represent the drive towards a positive outcome. Nevertheless, the fragility during survivorship can take over again: the lower positive beliefs after 2 years about one’s own future can be related to the high fear of cancer recurrence (FCR) defined as fear, worry, or concern about cancer returning or progressing [19,20]. Crist et al. [21] highlighted the FCR as being consistently associated with increased functionality impairment, psychological distress, stress response symptoms, and lower quality of life over time. Our findings confirmed this trend and proved that some specific points in time after primary care could be more related to emotional fragility and associated with recurrence. 

In conclusion, our study suggests the need to increase the clinical psychology service in terms of scheduled and tailored psychological screening about FCR, distress, and metacognition, and the provision of cancer-specific treatment for improved emotional resilience. The number of people surviving cancer is higher than ever before. However, many survivors fear a relapse even long after they have finished their course of treatment. Considering the positive results of this study, such fear-reducing intervention will pave the way for its wider availability to patients. 

Limitations of the study include reduced sampling and an observational study design. The analyses were observational only. Hence, we cannot eliminate the possibility that women who are distressed or depressed are more likely to perceive their breast cancer as more intrusive. The psychological battery was a self-report measurement of individual psychological symptoms and not measures of clinical indicators, such as digital data from remote daily detection. Furthermore, the relatively small sample size (as well as the distribution into six subgroups according to the time following diagnosis) limits the generalisability of our findings. One more limitation is the generalisability of results by cultural and social background: the study was conducted on the Italian population and it may have biased the findings. 

## 5. Clinical Implications

Patients with breast cancer are a vulnerable population with emotional needs that are impacted by age, the timing within the course of the disease, and clinical treatments. A combination of scheduled and tailored psychological screening during survivorship and the provision of tailored psychological treatments over that time might increase and enhance healthy outcomes not only in terms of the clinical path, but also in terms of reintegrating into social/work contexts with restored patients who are not weak and in need of support. Overall, our results provide empirical support for clinical practice to understand better and, consequently, respond efficiently to the needs of patients immediately after a breast cancer diagnosis. 

## Figures and Tables

**Table 1:** Demographic data of participants in the study.

	Young (N.36)	Adult (N.36)	Total (N.72)
	M 44 Sd ± 5	M 53 Sd ± 2	M 48 Sd ± 6
Education			
Not a graduate	13.90%	22.20%	18.10%
High school	47.20%	38.90%	43.10%
Graduate	30.60%	30.60%	30.60%
Relationship status			
Married/living with partner	75%	61.10%	68.10%
Single	19.40%	13.90%	16.70%
Widow	0	16.70%	8.30%
Occupation			
Housewife	25%	30.60%	27.80%
Employed	38.90%	25%	31.90%
Self-employed	33.30%	36.10%	34.70%
Treatments *			
Mastectomy	55.60%	27.80%	41.70%
Lumpectomy	44.40%	58.30%	51.40%
Chemotherapy	27.80%	44.40%	36.10%
Radiation therapy	63.90%	47.20%	55.60%
Hormonal therapy	63.90%	55.60%	59.70%
No treatment	8.30%	2.80%	5.60%
Cancer stage			
0	5.60%	2.80%	4.20%
I	25%	19.40%	22.20%
II	27.80%	19.40%	23.60%
III	2.80%	5.60%	4.20%
Timing			
T0 (6–11 M)	33.30%	27.80%	30.60%
T1 (12–18 M)	30.60%	27.80%	29.20%
T2 (19–24 M)	13.90%	13.90%	13.90%
T3 (25–31 M)	22.20%	22.20%	22.20%

* Treatments are not mutually exclusive.

**Table 2:** The mean (and standard deviations) of the raw scores performed in the psychological tests.

Psychological Tests	Age Groups					
	Young		Adult		Total	
	*X*	*Sd*	*X*	*Sd*	*X*	*Sd*
PDI	28.1 ± 6		25.9 ± 5.8		27 ± 6	
MCQ-30						
POS	11 ± 5		10 ± 4		10 ± 4	
NEG	15.8 ± 3.9		13.5 ± 3.3		14.6 ± 3.8	
CC	9.8 ± 4.2		10.6 ± 4.9		10.2 ± 4.5	
NC	11.8 ± 4.6		10.9 ± 3.3		11.3 ± 4	
CSC	15.5 ± 3.7		14.8 ± 3.8		15.4 ± 3.8	
TOT	64.1 ± 16.5		59.9 ± 14.6		62 ± 15.6	
DASS-21						
Depression	8.1 ± 6.5		6.9 ± 7.4		7.5 ± 6.9	
Anxiety	9.8 ± 9.1		5.4 ± 5.9		7.6 ± 7.9	
Stress	14.1 ± 7.6		11.3 ± 6.4		12.7 ± 7.1	

**Table 3:** Mean and standard deviations of the MCQ-30 sample distributed by age group and timing of evaluation.

MCQ-30	Young					Adult				
	**T0**	**T1**	**T2**	**T3**	**TOT**	**T0**	**T1**	**T2**	**T3**	**TOT**
	**X**	**X**	**X**	**X**	**X**	**X**	**X**	**X**	**X**	**X**
	**DS**	**DS**	**DS**	**DS**	**DS**	**DS**	**DS**	**DS**	**DS**	**DS**
**NEG**	14.5 ± 3.5	15.3 ± 4.1	14.4 ± 3.8	19.1 ± 2.9	15.8 ± 3.9	12.7 ± 2.5	14.5 ± 3.7	13.4 ± 4.8	12.8 ± 3.5	13.5 ± 3.3
**CC**	9.4 ± 2.6	8.8 ± 3.5	7.4 ± 1.1	13.3 ± 6.3	9.8 ± 4.2	8 ± 2.8	10.9 ± 5.2	14.6 ± 6.2	10.3 ± 4.2	10.6 ± 4.9
**NC**	11.8 ± 4	11.4 ± 5.7	9 ± 3.2	14.1 ± 4.4	11.8 ± 4.6	9.8 ± 3.1	11.1 ± 3.3	11.8 ± 2.4	10.9 ± 4.5	10.9 ± 3.3
**CSC**	15.2 ± 2.6	15.8 ± 5.1	14.8 ± 1.5	18 ± 3.5	15.9 ± 3.7	14.9 ± 3.8	16.3 ± 2.7	16 ± 5.5	13.5 ± 3.3	14.8 ± 3.8
**POS**	9.6 ± 3.9	11.7 ± 4.7	8.2 ± 3.0	14.1 ± 4.7	10.7 ± 4.5	8.3 ± 3.1	11.7 ± 4.0	9.6 ± 5.1	11.5 ± 4.2	10.4 ± 4.0

**Table 4:** Pearson *r* correlations between MCQ-30, DASS-21, and PDI scores for the sample.

Variables	DASS-21	DASS-21	DASS-21 Depression	PDI
	Stress	Anxiety		
**MCQ-30 POS**	0.158	0.167	0.088	0.188
**MCQ-30 NEG**	0.329 **	0.385 **	0.269 *	0.392 **
**MCQ-30 CC**	0.177	0.113	0.193	0.232 *
**MCQ-30 NC**	0.183	0.253 *	0.129	0.159
**MCQ-30 CSC**	0.149	0.163	0.021	0.113

* *p* > 0.01, ** *p* > 0.05.
